# Supplementary material for: Propagation of THz irradiation energy through aqueous layers: Demolition of actin filaments in living cells
Source: Sci Rep. 2020 Jun 2;10:9008. doi: 10.1038/s41598-020-65955-5 (PMC7265563; doi:10.1038/s41598-020-65955-5)
Supplement: Supplementary file 1 — Supplementary information. [file 41598_2020_65955_MOESM1_ESM.docx]

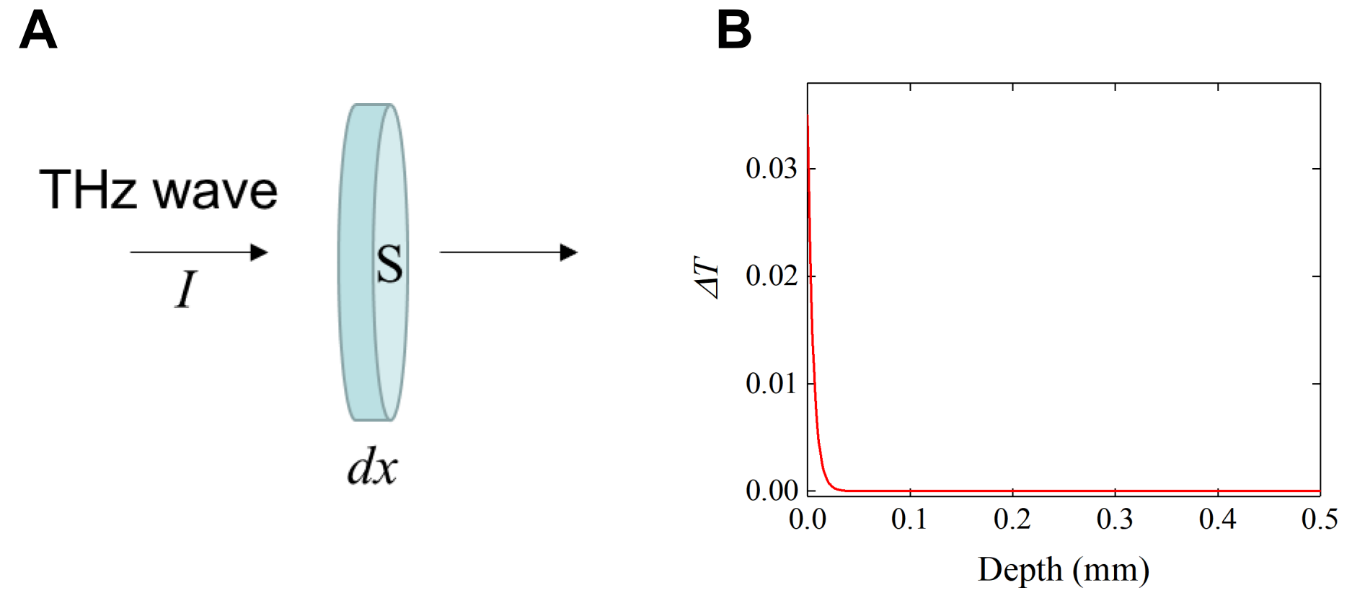
Supplementary Materials

**Figure S1. Estimation of THz heating effect with adiabatic model.**

(A) The model of THz wave irradiated to the water with minute thickness. (B) *ΔT* just after a single micro pulse of 80 μJ/cm^2^ irradiated to the water.


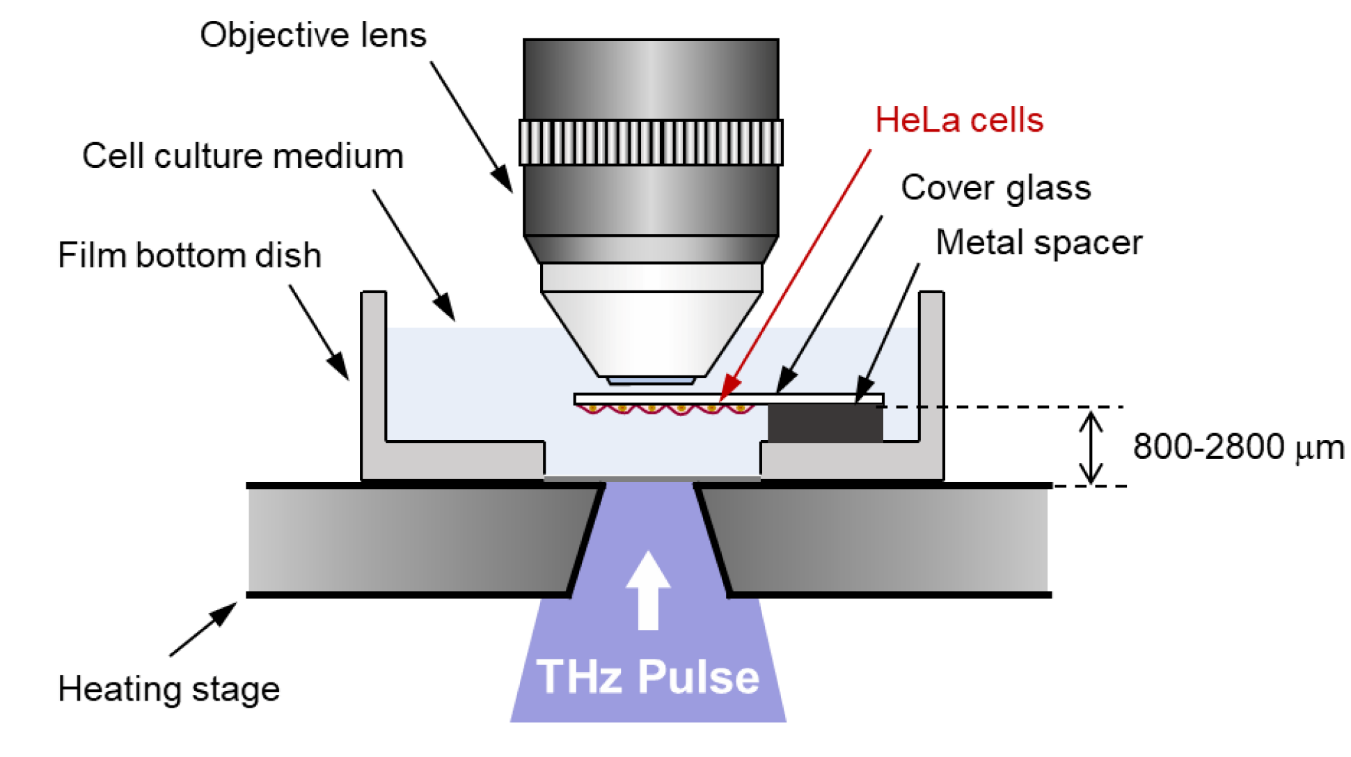
**Figure S2. Experimental set-up of cells in culture medium.**

HeLa cells were seeded on a cover glass and immersed in culture medium. The culture medium was kept at 37 °C during the experiment, and THz pulses were applied from the bottom of the dish.
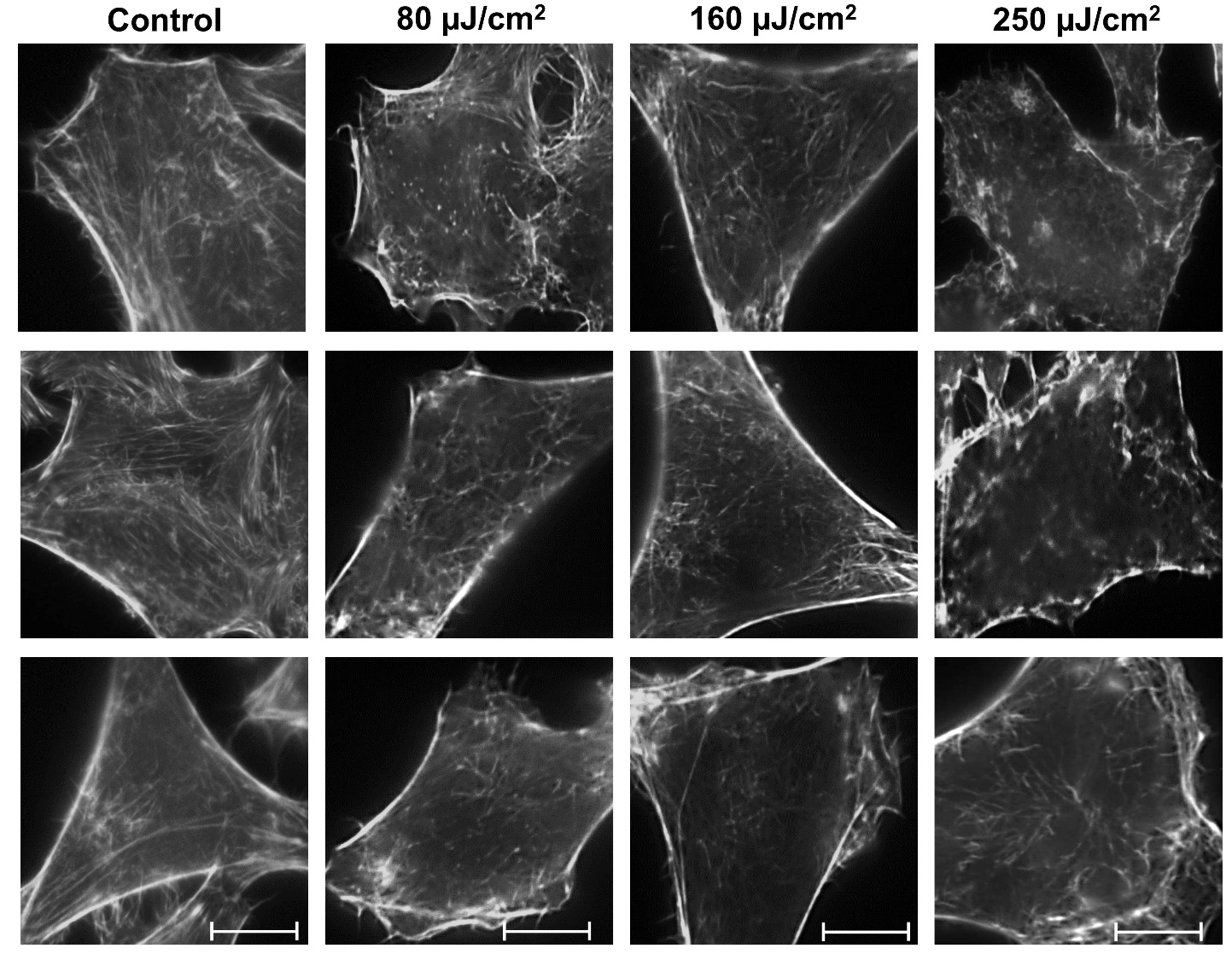
 **Figure S3. Immunofluorescence images of the cell cortex.**

After THz irradiation with the indicated energies for 30 min, HeLa cells were fixed and stained with AlexaFluor 594-phalloidin. Images were observed using spinning-disk confocal microscopy. The bar shows a scale of 10 µm.


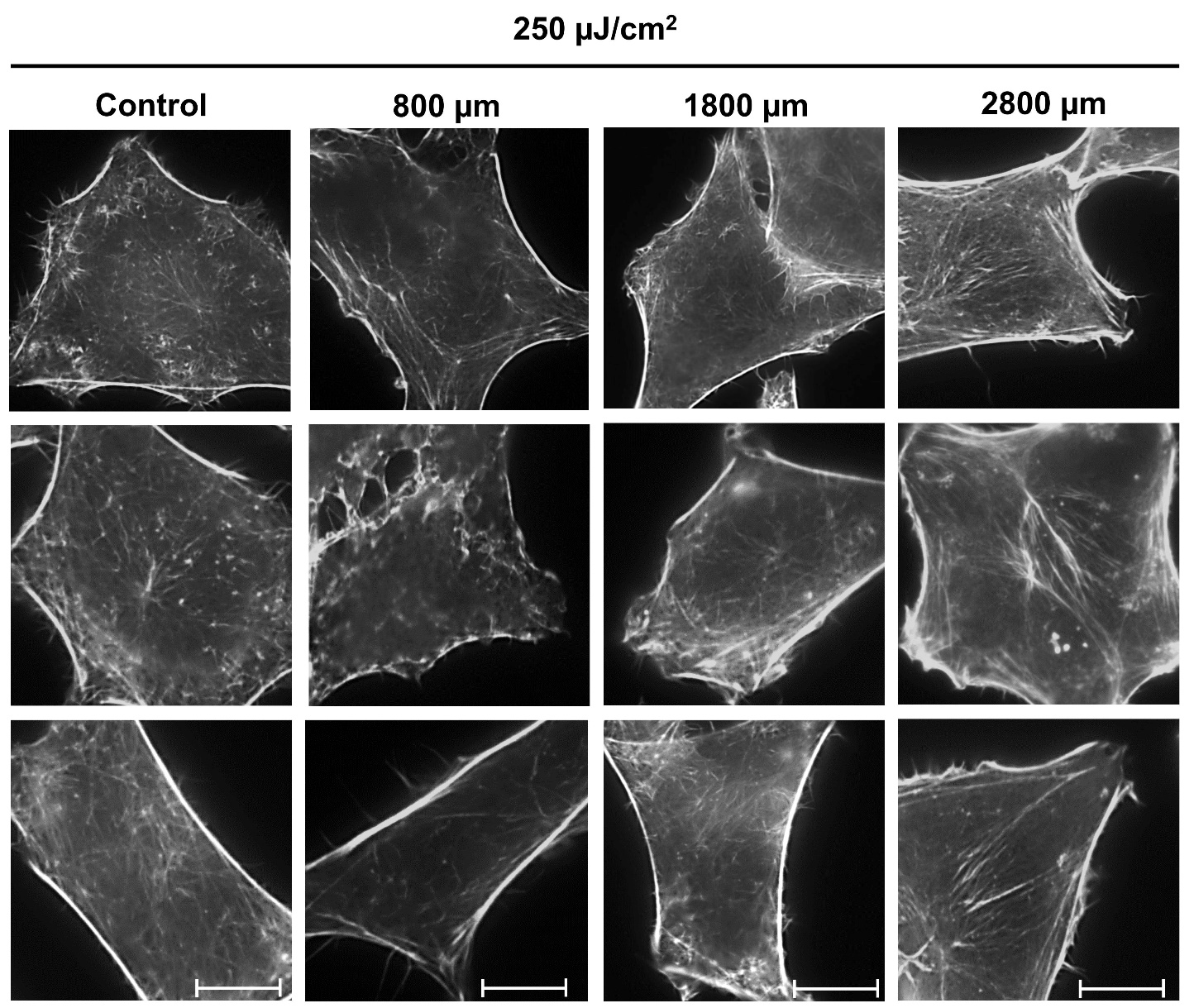
 **Figure S4. Immunofluorescence images of the cell cortex.**

After THz irradiation at the indicated distances for 30 min, HeLa cells were fixed and stained with AlexaFluor 594-phalloidin. Images were observed using spinning-disk confocal microscopy. The bar shows a scale of 10 µm.


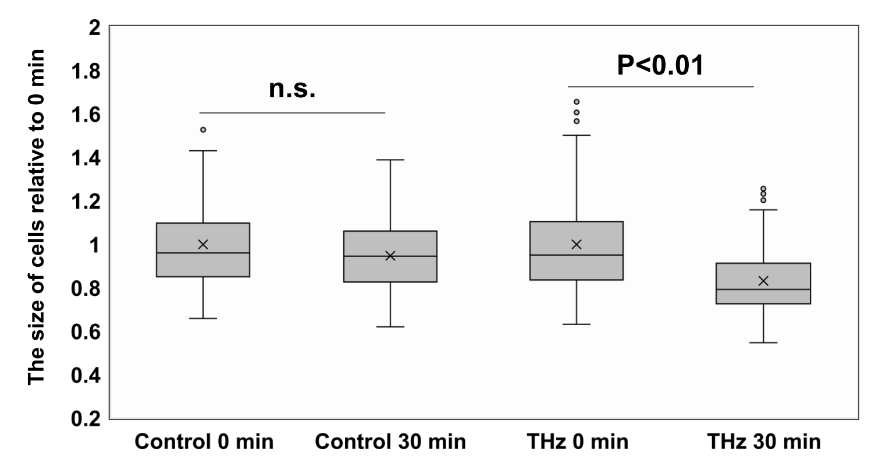


**Figure S5. Comparison of cell size before and after THz irradiation.**

The size of cells was measured in microscopy images using Image J software. The average cell area at 0 min was defined as 1.0. N >80 cells/group.


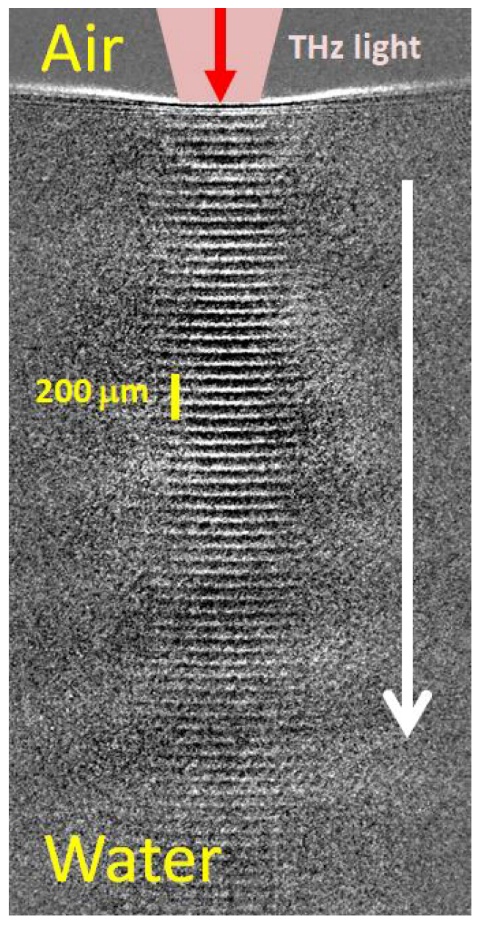


**Figure S6. THz-FEL light induced Shockwaves.**

Snapshot image of a train of shockwaves captured by an image-intensified CCD camera with a time gate of 10 ns. The vertical bar shows a scale of 200 µm.
